# Parkinson’s: a syndrome rather than a disease?

**DOI:** 10.1007/s00702-016-1667-6

**Published:** 2016-12-27

**Authors:** Nataliya Titova, C. Padmakumar, Simon J. G. Lewis, K. Ray Chaudhuri

**Affiliations:** 1 Federal State Budgetary Educational Institution of Higher Education, N.I. Pirogov Russian National Research Medical University, Ministry of Healthcare of the Russian Federation, Moscow, Russia; 2 Parkinson’s Disease Service for the Older Person, Rankin Park Centre, John Hunter Hospital, HNELHD, Newcastle, NSW Australia; 3 Brain and Mind Centre, University of Sydney, NSW, Australia; 4 National Parkinson Foundation International Centre of Excellence, Kings College and Kings College Hospital, London, UK; 5 National Institute for Health Research (NIHR) Mental Health Biomedical Research Centre (BRC) and Dementia Unit at South London and Maudsley NHS Foundation Trust, London, UK

**Keywords:** Parkinson’s disease, Parkinson’s syndrome, Non-motor symptoms, Non-motor subtypes, Individualized medicine, Neurotransmitter

## Abstract

Emerging concepts suggest that a multitude of pathology ranging from misfolding of alpha-synuclein to neuroinflammation, mitochondrial dysfunction, and neurotransmitter driven alteration of brain neuronal networks lead to a syndrome that is commonly known as Parkinson’s disease. The complex underlying pathology which may involve degeneration of non-dopaminergic pathways leads to the expression of a range of non-motor symptoms from the prodromal stage of Parkinson’s to the palliative stage. Non-motor clinical subtypes, cognitive and non-cognitive, have now been proposed paving the way for possible subtype specific and non-motor treatments, a key unmet need currently. Natural history of these subtypes remains unclear and need to be defined. In addition to in vivo biomarkers which suggest variable involvement of the cholinergic and noradrenergic patterns of the Parkinson syndrome, abnormal alpha-synuclein accumulation have now been demonstrated in the gut, pancreas, heart, salivary glands, and skin suggesting that Parkinson’s is a multi-organ disorder. The Parkinson’s phenotype is thus not just a dopaminergic motor syndrome, but a dysfunctional multi-neurotransmitter pathway driven central and peripheral nervous system disorder that possibly ought to be considered a syndrome and not a disease.

## Background

In 1817, James Parkinson, the English physician, described a syndrome and he named Paralysis Agitans (An Essay on the Shaking Palsy), which was subsequently termed Parkinson’s disease (PD) by Jean-Marie Charcot in view of his initial description. (Parkinson 1817) Despite including significant details regarding many of its key non-motor symptoms including sleepiness, fatigue and dysautonomia over the years, PD has almost become synonymous with a dopamine deficiency motor syndrome. Clearly, this position has been reinforced by the dramatic effect of levodopa in relieving the motor features of PD, which whilst revolutionizing the outlook for patients has fallen well short in addressing the non-motor syndrome (NMS) (Langston 2006). This deficiency in clinical practice is not surprising when one considers that it was not until the early 2000’s that the first validated tools to comprehensively evaluate the complex medley of NMS in PD were developed (Chaudhuri et al. 2006). These objective measures laid bare the extent of the NMS and its major impacts on quality of life in PD (Martinez-Martin et al. 2011), as well as highlighting the need for specific focused non-motor therapies (Schrag et al. 2015b).

The multi-focal and multi-neurotransmitter driven pathology of PD has been emphasized by the landmark work of Braak et al. who suggested that a “bottom-up” six-stage pathological process could account for most cases studied neuropathologically (Braak et al. 2003). This work has proposed that alpha-synuclein pathology critically resulting in neuronal Lewy body deposition and cell death spreads from regions interfacing with the “environment”. In this model, regions including the olfactory bulb and the enteric nervous system with its connections to the medulla through the dorsal motor nucleus of the vagus nerve act as a ‘conduit’ for a spreading pathology perhaps mediated by a prion-like process (Klingelhoefer and Reichmann 2015). Such a model allows both dopaminergic and non-dopaminergic populations to be differentially affected and in so doing may permit insights into the motor and NMS. Whilst by no means perfect, this concept of a multi-neurotransmitter, multi-organ (brain and peripheral nervous system) disorder is now well established in the literature with supportive pathological and biomarker driven projects (Jellinger 2015; Sauerbier et al. 2016). A multitude of factors are now thought to underlie the final clinical expression of the complex disorder that is PD and some are listed in Table 1. All of above would suggest that PD is a heterogeneous syndrome defined by a variable collection of signs and symptoms that manifest to differing extents within individual patients. However, we are conscious of the fact that some would argue that robust evidence base for PD being a multisystem disorder with heterogenic genetic basis is lacking. We consider these facts and provide evidence to support the syndromic nature of Parkinson’s and debate whether Parkinson’s should be called a disease or a syndrome. We will thus use the term Parkinson’s disease (PD) interchangeably with Parkinson’s syndrome in this paper.

**Table 1:** List of proposed mechanisms and pathophysiological basis for the expression of clinical signs of Parkinson’s disease

Genetics and epigenetics
LRRK2, GBA mutations, and higher rates of PD in certain ethnic groups, such as Ashkenazi Jews, Inuit populations
Dietary or occupational exposure to organic toxins (insecticides for example)
Gene interaction with environment (higher risk in agricultural communities, lower risk in smokers, head trauma)
Alpha-synuclein abnormalities
Misfolding, oligomeric form, and altered proteostasis and neurotoxicity
Susceptibility of ageing brain
Synaptic dysfunction and loss of synaptic level functioning
Prion-like intra axonal transport (gut to brain)
Amyloid and Tau deposition particularly in older PD and dementia
Mitochondrial dysfunction (reduced complex 1 activity)
Oxidative stress causing cell damage and death
Neuroinflammation which may trigger misfolding of alpha-synuclein
Altered gut microbiota and reduced mucin increasing gut permeability and possible inflammatory spread to brain
Neurotransmitter linked abnormalities (selective or in combination as detailed in the paper)
Alteration in cerebral functional network and signaling function
Adenosine receptor abnormalities

## Pathological and neurotransmitter basis of the Parkinson’s syndrome: it is not all dopamine

**Fig. 1:**
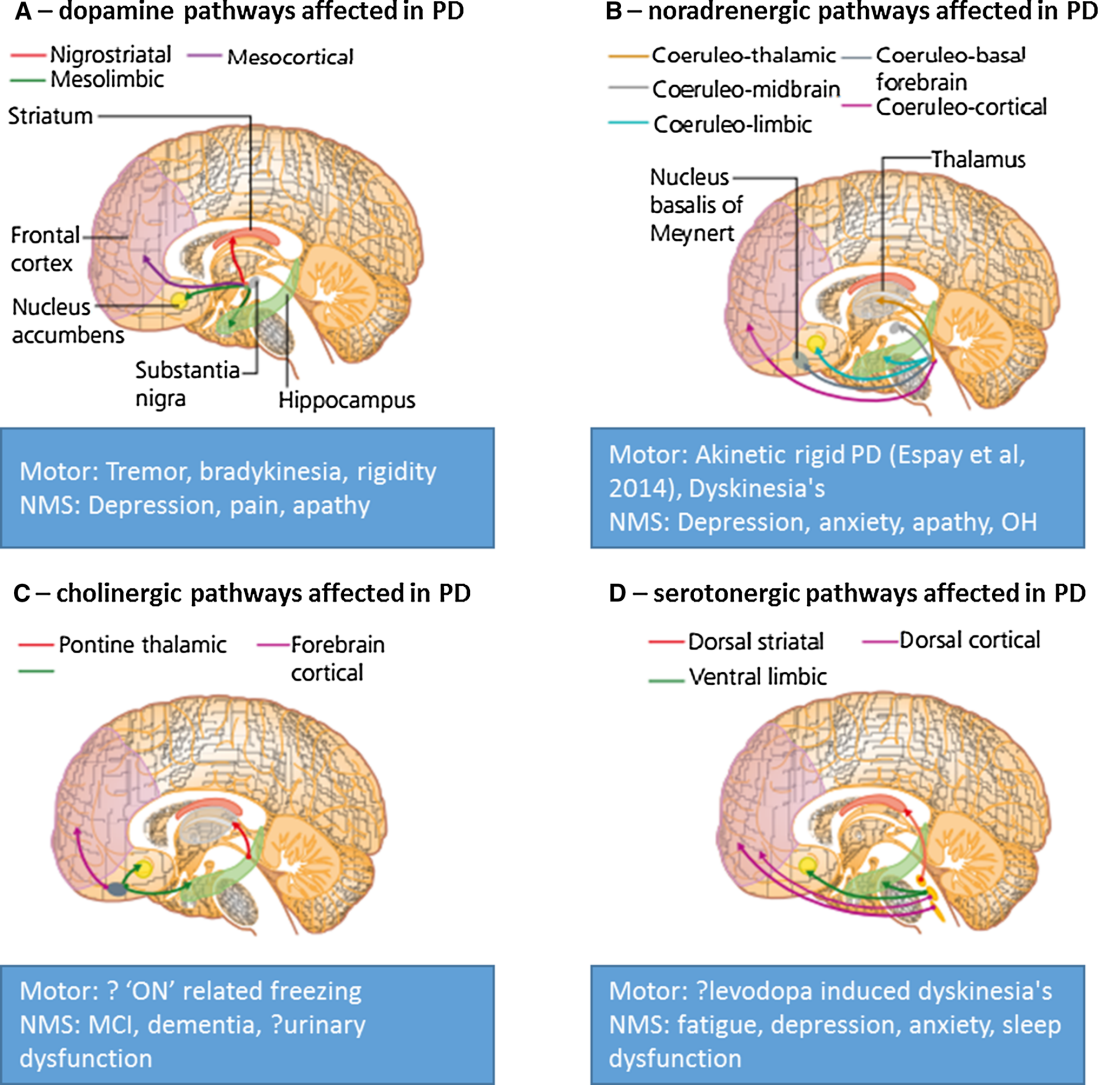
Multi-system and multi-neurotransmitter dysfunction in PD. NMS non-motor symptoms; *OH* orthostatic hypotension, *MCI* mild cognitive impairment Adapted from Chaudhuri and Fung (2016)

The clinical phenotype of PD is variable and a wide range of NMS underpin both the prodromal and clinical stages of PD (Schrag et al. 2015a; Zis et al. 2015). These NMS range from sleep dysfunction, such as rapid eye movement behavior disorder (RBD) to cognitive dysfunction and apathy, which may arise from a varying density of Lewy body deposition and non-dopaminergic patterns of neurodegeneration in PD (Jellinger 2012) and response to medications. The heterogeneity of Parkinson’s is also underpinned by a complex pathophysiology which ranges from misfolding of alpha-synuclein to amyloid and tau protein deposition, neuroinflammation, mitochondrial dysfunction, genetic and epigenetic factors, as well as the brainstem origin of the condition. The clinical phenotypic variations, therefore, represent the consequence of widespread brain and peripheral Lewy body pathology and not a single neuronal structure, such as the substantia nigra or isolated loss of the dopamine neurotransmitter system (Jellinger 2012; Todorova et al. 2014). The neurotransmitter systems affected are widespread and the convergence of deficits in multiple transmitter pathways which include the dopaminergic, cholinergic, noradrenergic, and serotonergic systems among others. These possibly drive abnormal central brain network activity which results in the clinical expression of the highly heterogeneous Parkinson syndrome (Fig. 1).

## Selective vulnerability of non-dopaminergic neurons

The Braak hypothesis of alpha-synuclein accumulation starting in the lower medulla and the anterior olfactory bundle with a subsequent spread via pons to the midbrain would potentially affect a range of non-dopaminergic nuclei along the route, including the locus coeruleus and the raphe area, even before there was any significant involvement of substantia nigra (Braak et al. 2003). A number of authors have reported that non-dopaminergic nuclei may degenerate at a faster rate and sometimes to a greater degree than dopaminergic neurons in the early and prodromal stages of PD. Indeed, a number of studies have reported that there may be a greater loss of cholinergic pedunculopontine nucleus neurons and substance P—containing neurons in dorsal motor nucleus of the vagus (over 70%) with relative sparing (<5%) of tyrosine hydroxylase-immunoreactive neurons in the dopaminergic system (Hirsch et al. 1987; Jellinger 1987; Halliday et al. 1990). In addition, Jellinger (2012) has also shown that neuronal loss in the dorsal motor nucleus of vagus (DMV) could be as profound as that in the substantia nigra (SN). It is well accepted that the DMV is a one of main centers for autonomic signaling and may be responsible for autonomic symptoms, such as constipation, which is commonly seen in prodromal stage of PD. Differences in the onset age of PD (i.e., late versus early) may also imply a brainstem pathology dominant clinical picture in the early onset disease, while in late onset disease, brainstem pathology is associated with cortical Lewy body deposition. The reasons underlying this age-related discrepancy are not resolved but may relate to issues of depleted neural reserve and immune-competence (Halliday et al. 2011).

## Peripheral involvement in PD

**Fig. 2:**
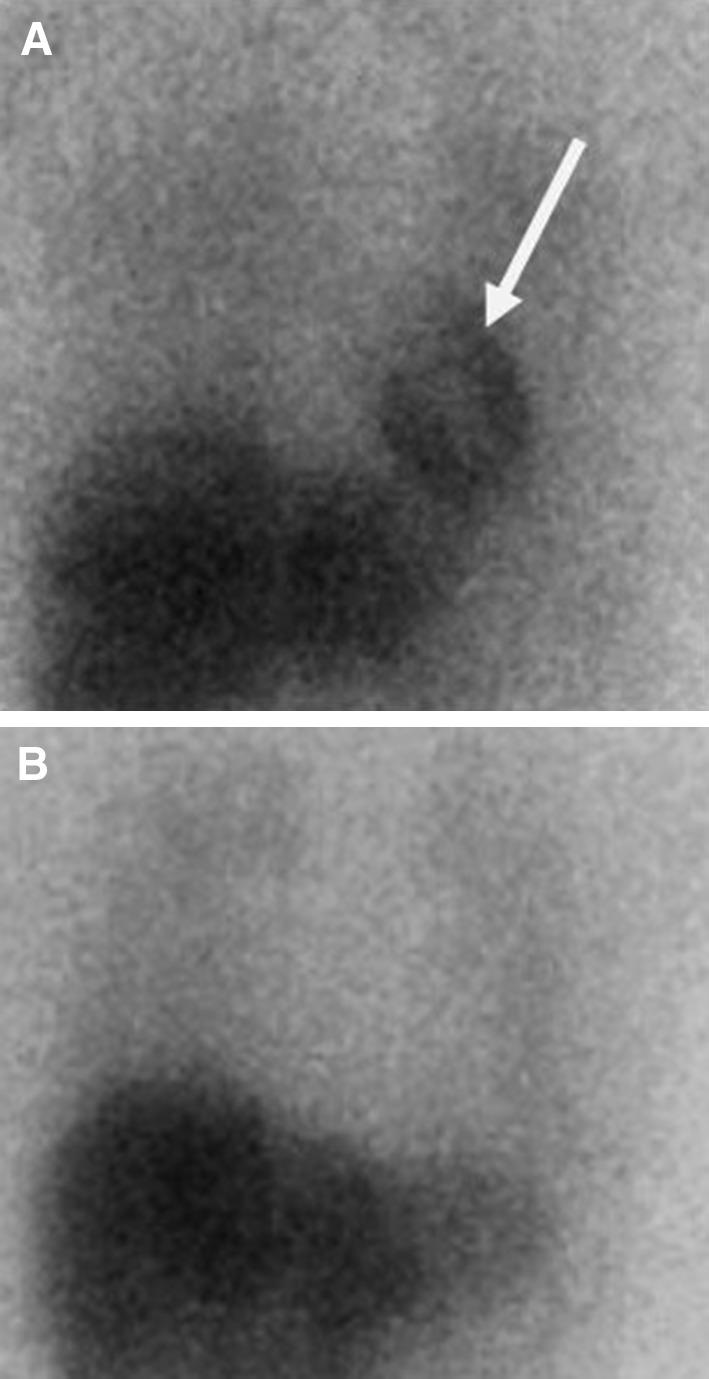
Cardiac meta-(123)I-iodobenzylguanedine (MIBG) imaging. **a** Shows a subject with normal visualisation of the heart (*arrowed*), while **b** shows non-visualisation of the heart as in PD as evidence of postganglionic peripheral sympathetic dysfunction Picture courtesy nuclear imaging department, Kings College Hospital, London

The syndromic nature of PD is also evident in the fact that there is pathological involvement of many peripheral organs as discussed briefly below. For instance, the central noradrenergic system is prominently involved in the neuropathology of PD. In addition, noradrenaline is recognized as a key transmitter in the sympathetic nervous system, which in particular innervates the vascular system and the heart. Using in vivo cardiac meta-(123)I-iodobenzylguanedine (MIBG) or PET 18F-fluorodopamine (FD), imaging has demonstrated the loss of noradrenergic cardiac innervation in patients with early PD (Fig. 2). Further evidence of possible prodromal cardiac sympathetic involvement is suggested by work conducted in asymptomatic brain donors who had incidental Lewy body disease (i.e., potentially a prodromal stage of PD) who show a reduction of tyrosine hydroxylase-containing axons in the myocardium of the heart (Iwanaga et al. 1999).

**Fig. 3:**
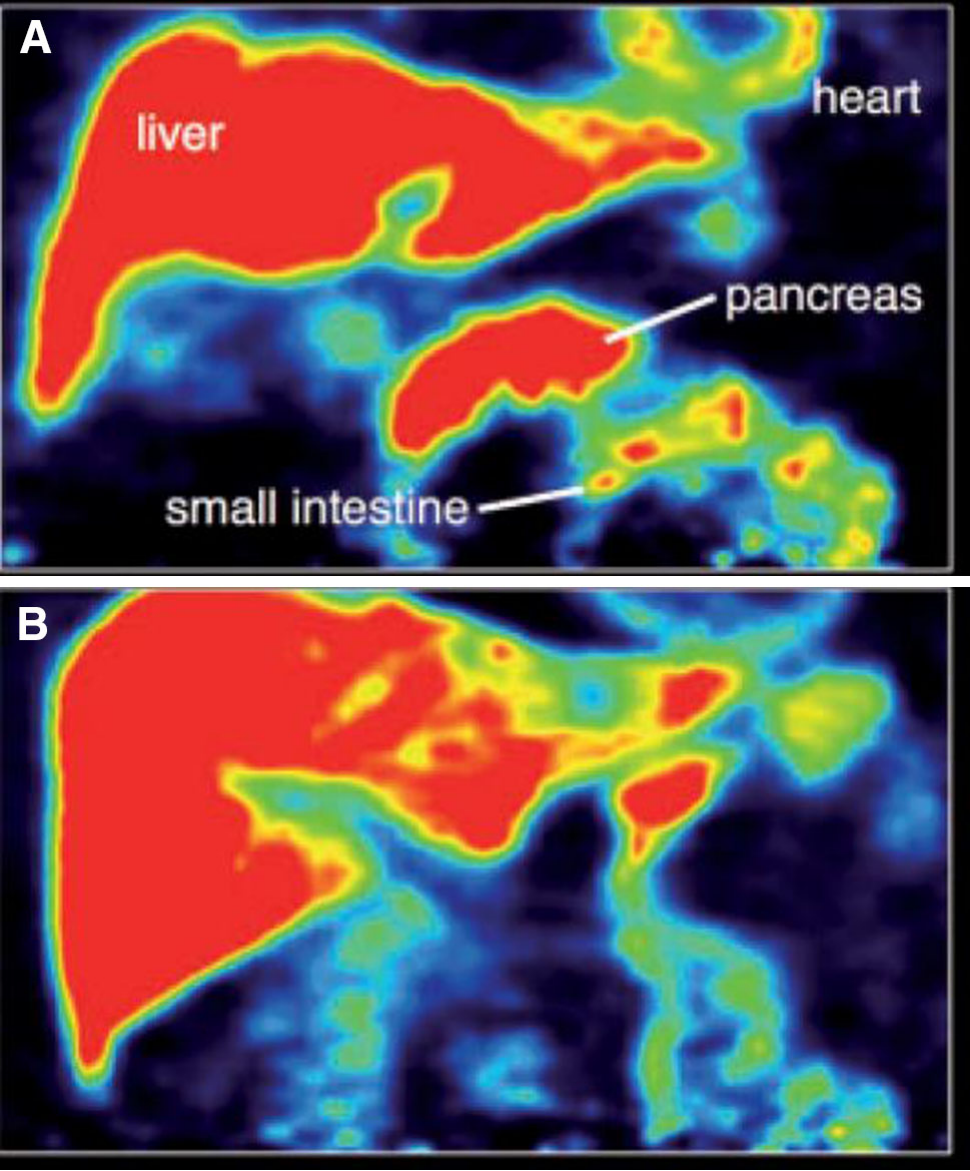
5-[11C]-methoxy-donepezil PET-imaging in normal subjects (**a**) and Parkinson’s disease (**b**). **b** Showing reduced uptake of pancreas and intestine Taken from Gjerløff et al. (2015)

**Fig. 4:**
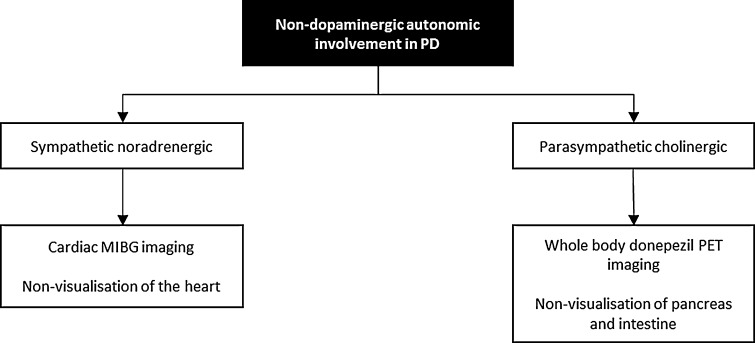
Peripheral sympathetic and parasympathetic dysfunction of the Parkinson’s syndrome as shown by in vivo imaging. *MIBG* meta-(123)I-iodobenzylguanedine, *PET* positron emission tomography

In addition to these sympathetic noradrenergic deficiencies, recent in vivo imaging with 5-[11C]-methoxy-donepezil has made it possible to measure peripheral acetylcholinesterase density a marker of parasympathetic function. Work in PD using this approach has shown significantly decreased 11C-donepezil binding in the small intestine and pancreas of PD stage patients (Fig. 3) (Gjerløff et al. 2015). This data suggests that the pathophysiology of PD also involves the parasympathetic innervation (Fig. 4).

**Fig. 5:**
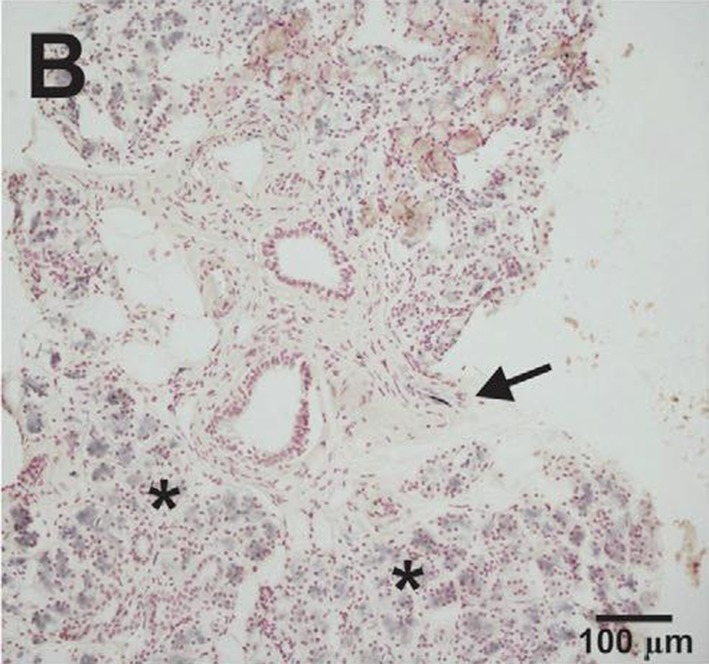
Submandibular gland needle biopsy from a subject with PD with immune-histochemical staining for phosphorylated alpha-synuclein showing positive inclusion. *Arrow* points to an immunoreactive nerve fiber within a stromal nerve fascicle. *Asterisks* indicate nonspecific immunoperoxidase staining of gland cell cytoplasm Taken from Adler et al. (2016)

Adler et al. (2016) have shown that submandibular gland needle biopsies were able to identify phosphorylated alpha-synuclein staining in 74% of the early PD subjects (Fig. 5), whilst other research teams have described phosphorylated alpha-synuclein deposition across the myenteric plexus, submucosal layer as well as the mucosal nerve fibers of the intestine (Shannon et al. 2012). Involvement of skin with accumulation of alpha-synuclein has also been described (Gibbons et al. 2016).

## The clinical translation: subtypes

The diagnostic concept of Parkinson’s disease is changing and an ongoing revision of its diagnostic criteria by the International Movement Disorders Society has included a range of non-motor symptoms (NMS) as part of the core parameters (Postuma et al. 2015). This would suggest that there is a greater awareness of the clinical heterogeneity of PD, which is no longer viewed as a disease with motor features alone. The recognition mixed motor and non-motor phenotypes have been well documented in the literature and many initial studies attempted to understand these variances through a “matched groups” approach with classifications based on predetermined patient attributes, such as age of disease-onset, cognitive performance, motor phenotype, and disease severity. However, all of these approaches suffer from the limitations arising from the prospective assumptions about the classification, namely the arbitrary division of patients based on the criteria adopted. To avoid this, more recent work has sought to utilize data-driven methodologies, such as cluster analysis.

One recent systematic review of the cluster analyses performed in PD has revealed that subgroups do appear to exist and that there is a common division occurring between a milder younger onset and a more aggressive older onset phenotype (van Rooden et al. 2010). Results obtained from individual studies have highlighted the existence of four distinct PD subgroups, namely (1) younger disease-onset, (2) tremor dominant, (3) non-tremor dominant, and (4) rapid disease progression (Lewis et al. 2005; Reijnders et al. 2009; Selikhova et al. 2009). These initial studies highlighted that whilst tremor dominant patients have relative NMS sparing, the non-tremor dominant subgroup is more associated with cognitive impairment and mood disturbance. Indeed, more recent work has identified a differential expression of mild cognitive impairment across these subgroups, with the highest frequency observed in the non-tremor dominant cluster, which was also associated with a higher prevalence of freezing of gait, hallucinations, daytime somnolence, and RBD compared with other subgroups (Szeto et al. 2015).

To avoid the impact of dopaminergic therapy, some authors have performed cluster analysis in untreated PD patients, although there are still problems with this approach. Using this strategy, motor and several non-motor symptom dominant clusters have been identified in two large studies (Erro et al. 2013; Pont-Sunyer et al. 2015), whilst other observers have attempted to clinically define the non-motor clusters to specific non-motor subtypes of PD (Sauerbier et al. 2016; Marras and Chaudhuri 2016). Heterogeneity is again evident in these analyses, and for instance, Erro et al. (2015) reported that their non-motor dominant cluster had urinary dysfunction, which predicted a rapid progression rate of the motor syndrome of PD. In the ONSET-PD study, specific non-motor PD clusters, which ranged from cognitive and mood clusters to sensory, RBD dominant, and autonomic dysfunction-related clusters were reported which tallied well with the NMS dominant subtypes described by Sauerbier et al. (2016). Indeed, biomarker driven studies have now shown evidence that these subtypes can be further defined by specific neurochemical dysfunction, at least in part, suggesting that in future, progression pattern of these specific NMS subtypes could be examined.

**Fig. 6:**
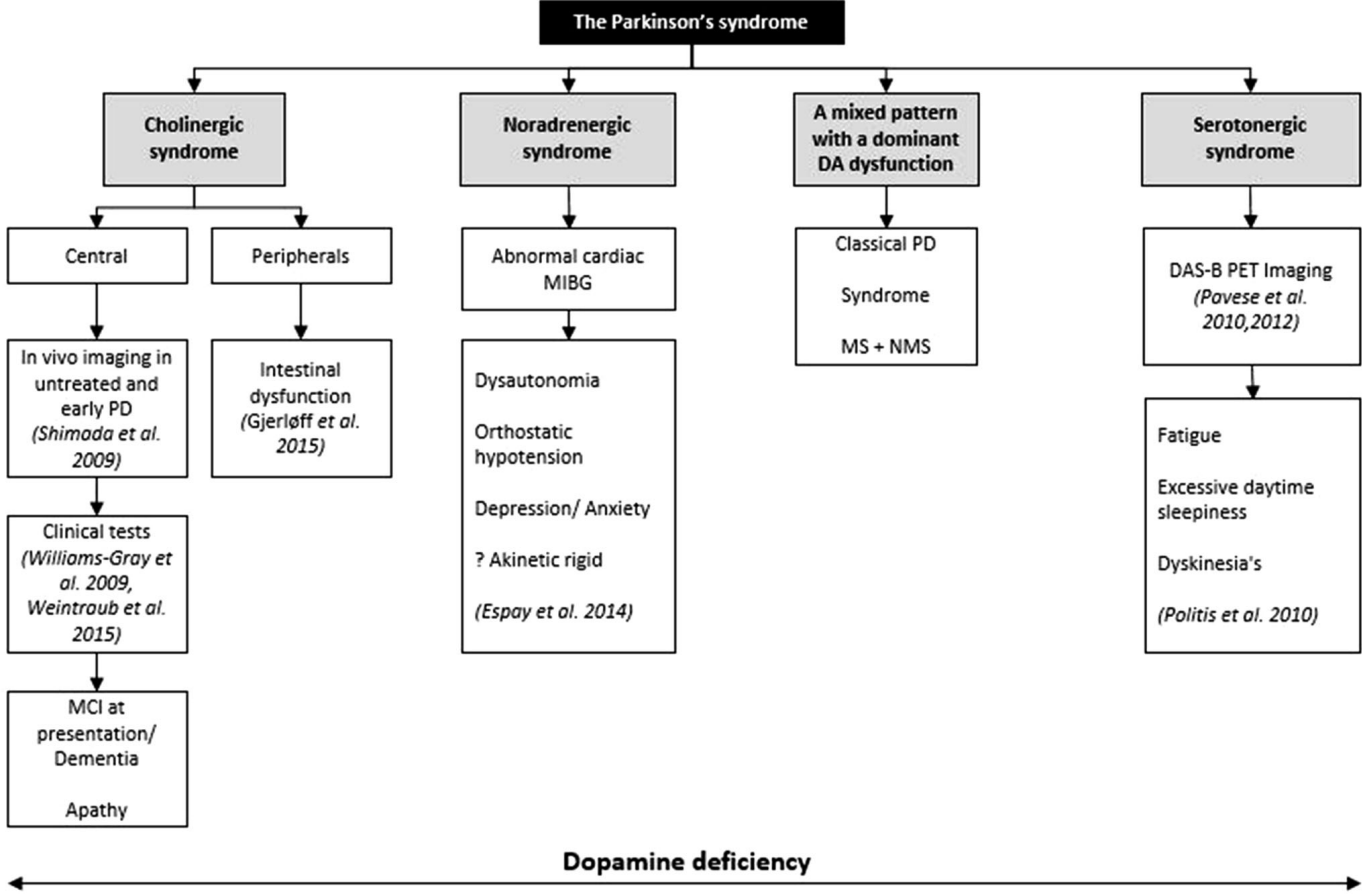
Proposed flowchart showing the various selective (does not exclude overlap) neurotransmitter pathway dysfunction as delineated by in vivo imaging or clinical tests and the resulting clinical phenotype of NMS dominant subtypes. Dopamine deficiency underpins the whole condition. The imaging correlates could emerge as possible biomarkers in future MS motor syndrome. NMS non-motor syndrome

It has been suggested that NMS subtypes may be more stable over time compared to the motor subtypes PD as the former is underpinned by specific patterns of neurotransmitter pathway dysfunction (Marras and Chaudhuri 2016). A proposed example is offered in Fig. 6.

## Possible clinical consequences

Identification of specific NMS subtypes may, in future, help fashion more personalized therapies and individualized medicine (Schrag et al. 2015b). For example, some might argue that a PD variant identified to have cognitive dysfunction at onset is likely to have a more cholinergic syndrome that would merit combined therapy with dopaminergic and cholinesterase inhibitors. Moore and Barker (2014) argue for robust multimodal biomarkers that may predict the development of PD dementia and help develop specific and individualized therapies. A stronger clinical “sleep” phenotype (Sauerbier et al. 2016) would possibly be underpinned by serotonergic raphe dysfunction and may, therefore, have a narcoleptic phenotype (Pavese et al. 2012; Ylikoski et al. 2015). In these patients, there might even be an abnormal sensitivity to dopamine D3 receptor agonists, which might, therefore, be preferably avoided (Sauerbier et al. 2016).

## Conclusion

It is crucial that the clinical heterogeneity of PD is better recognized as it is likely that multimodal biomarker methods will show specific patterns of underlying cerebral and extra-cerebral neurotransmitter dysfunction. Such findings would correlate well with specific clinical subtypes of PD, particularly in the newly emerging concept of non-motor subtypes. Clinically, the heterogeneity of PD is also reflected by broad overlap of PD with parkinsonian syndromes, such as dementia with Lewy bodies or parkinsonian variants of multiple system atrophy, which is reflected in the recent revision of the diagnostic criteria for PD (POstuma et al. 2015). We acknowledge that, at this time, there is no robust or convincing evidence base to suggest that PD is definitively a multisystem disorder. However, evidence provided in this review with consequent abnormalities of functional networks within the brain is likely to drive the heterogeneity of PD, with important implications for clinical translational and prognostic research for the future.
